# Synthesis of pure and C/S/N co-doped Titania on Al mesh and their photocatalytic usage in Benzene degradation

**DOI:** 10.1038/s41598-019-53189-z

**Published:** 2019-11-12

**Authors:** S. Modanlu, A. Shafiekhani

**Affiliations:** Department of Physics, Alzahra University, Vanak, Tehran 1993891167 Iran

**Keywords:** Pollution remediation, Photocatalysis, Nanoparticles, Synthesis and processing

## Abstract

Pure and co-doped Titania thin films were prepared on aluminum substrates through the sol-gel method. The co-doped sample showed higher photocatalytic activity on benzene degradation compared to pure TiO_2_ under visible light illumination. XRD results showed the anatase phase for both TiO_2_ and co-doped TiO_2_ lattices with an average crystalline size of 12.9 and 10.4 nm, respectively. According to the UV-visible absorption spectra results, co-doped Titania showed higher visible light absorption compared to pure Titania. The synergistic effect of dopants caused a redshift to visible light absorption and also the lifetime of the photogenerated electron-hole were increased by induced electron levels in Titania lattice. The novelty of this study is the reactor’s specific design. We employed Al mesh as thin film substrate for 3 main reasons, first, the large surface area of the Al mesh causes to increase specific surface area of the photocatalysts, also it is a formable substrate which can be engineered geometrically to decrease the shadow spots so the thin films will receive the highest light irradiation. Also, the Al mesh flexibility facilitates the procedure of reactor design to reach a minimum pressure drop of airflow while it is installed in the air conditioners or HVAC systems.

## Introduction

Nanotechnology as an important field of modern research deals with the synthesis and modification of particle structures. synthesis of nanoparticles is one of the most interesting studies in recent years due to their wide range of applications in different fields like medicine, biotechnology, material sciences, chemistry, physics, photocatalysis, electronics, and etc.^1,2^.

The growth of environmental pollution has become a global issue and many projects have been applied to solve this problem. There are more than 300 different types of indoor volatile organic compound*s* (VOCs) and benzene is known as the most dangerous VOCs. It is considerable that only 1 ppm of benzene may cause blood poisoning and cancer^3^.

Most of the people have indoor activity more than 19 hours per day. This fact causes a serious concern about the indoor air quality. Increasing of the indoor VOCs leads to some major issues such as sick building syndrome, headache, nausea, tiredness, and difficulty in concentration^4^.

Nevertheless there are several environmental treatment technologies like ionization, condensation and etc. All these methods may decrease indoor VOCs, but also may reduce the air quality while photodegradation of organic pollutants is known as an efficient, clean, and cost benefit alternative for environmental treatment^5,6^.

So far, numerous photocatalysts such as various oxide, sulfide semiconductors and polymers have been modified for photocatalytic usage. Moreover, between various semiconductors employed as photocatalyst, TiO_2_ is the most common photocatalyst for its simple but reliable synthesis methods, strong oxidation and charge carrier transportation, and resistance to photo-corrosion, low pollutant load, low toxicity, chemical stability and being cost benefit^7,8^.

TiO_2_ has been employed as photocatalyst in both aqueous and air media. However, due to the wide band gap of Titania (3.0–3.2 eV, UV region) only a small portion of solar spectrum can be used for photocatalytic applications^9^.

There are various methods to increase the performance of Ti- based photocatalysts in presence of visible light such as surface modification, metal ion or nonmetal ion doping, and coupling with other semiconductors with narrow band-gaps^10,11^.

Recently, co-doping is chosen as a promoting method for surface modification and nitrogen is used widely in coherence with other metals or nonmetals to shift absorption edge of TiO_2_ to the lower energies and improve photocatalytic activity^12–19^.

Here, we have reported the synthesis and surface modification of Titania thin films through dip coating method. Co-doping of nitrogen/carbon/sulfur as three nonmetal dopants is applied for surface modification. The non-metal dopants cause a redshift in TiO_2_ band gap to visible region. The recombination of electron-holes will be postponed through inducing of extra electron bands and also applying tri-doped Titania causes a composed of smaller particles or crystal sizes with more efficiency in degrading organic pollutants^20^.

The photocatalytic activity of as-prepared films is evaluated by benzene degradation. The substrate (Al mesh) and the design of the reactors are based on commercial purpose.

## Experimental Detail

### Thin films preparation

#### Sol gel

Here, TiO_2_ thin films were prepared via sol-gel procedure^21^. Titanium (IV) isopropoxide (TTIP, Ti[OCH(CH_3_)_2_]_4_, ≥97.0%, Sigma.) and anhydrous ethanol (ethanol absolute, 99.8% Merck) were used as Ti precursor and solvent, respectively. HCl (37%) was used for pH adjustment and distilled water was applied for hydrolysis process. Moreover, PEG (polyethylene glycol, 6000) and TEA (3- ethanolamine) were used as porosity agent and stability, respectively.

The first solution was prepared by dissolving 30 ml of TTIP in 200 mL anhydrous ethanol. Afterward, 7 ml of TEA and 1.6 gr of PEG were added to the solution under a vigorous stirring.

The second solution was prepared by a mixture of 200 ml ethanol, 1.15 ml of HCl, and 3 ml of distilled water. This solution was gently added to the first solution. The as-prepared sol was stirred overnight for aging purpose.

C/S/N co-doped Titania was prepared through the same procedure like pure TiO_2_. Thiourea was employed as C, S, and N dopants precursor. According to ref.^22^, 9 gr of Thiourea was added to the first solution and other steps were followed as the previous procedure.

#### Dip-coating process

Aluminum meshs were applied as TiO_2_ thin film substrates. All the substrates with the dimension of 10 × 10 cm^2^ were washed in an ultrasonic bath at 2 steps, first using distilled water then ethanol/acetone (1:1) solution for 20 minutes. The dried plates were dipped in the as-prepared sol. Then, the plates were pulled up with 9 cm/min after 5 minutes, dried at room temperature and in the oven with 90 °C for 20 and 30 minutes, respectively. The process was repeated four times to gain an appropriate thickness (about 3.5 μm) for photocatalysis applications. Finally, all samples were annealed at 450 °C in the furnace for 1 h.

#### Sample characterization

X-ray diffraction (XRD) patterns of catalysts were recorded via X’Pert Pro MPD X-ray diffractometer with Cu-Kα (λ = 0.15406 nm). The weight and atomic ratio of dopants in the TiO_2_ meshwork were characterized by energy dispersive X-ray (EDX, Phillips, XL30). The morphology of samples was specified using scattering electron microscope (KYKY-EM3200). UV–vis absorption spectra of samples were recorded trough spectrophotometer (StellarNet -EPP-2000) with scanning range of 200–800 nm.

### Evaluation of photocatalytic activity

**Figure 1:**
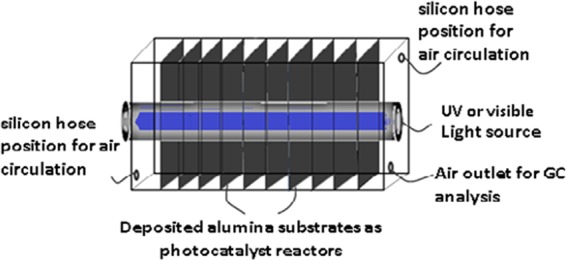
Schematic diagram of photocatalytic reactor.

Benzene was selected as a crucial VOC pollutant to evaluate the photocatalytic activity of the samples. In a typical experiment, 8 plates of pure Titania (or C/S/N-Titania) thin films were placed in an aluminum rectangle cube cell. Four fans (12 V) provided air circulation in the cell. One UV-black lamp (15 W) with the peak in 370 nm was installed in the center of the cell to produce UV irradiation. A same procedure was applied using an ordinary fluorescent lamp (15 W) as visible light source. In every run, 50 μg/lit of benzene was injected into the cell. Benzene concentration was checked trough gas chromatograph (YL-clarity 6500). The schematic diagram of the photocatalytic system is shown in Fig. 1.

## Results and Discussion

### SEM results

**Figure 2:**
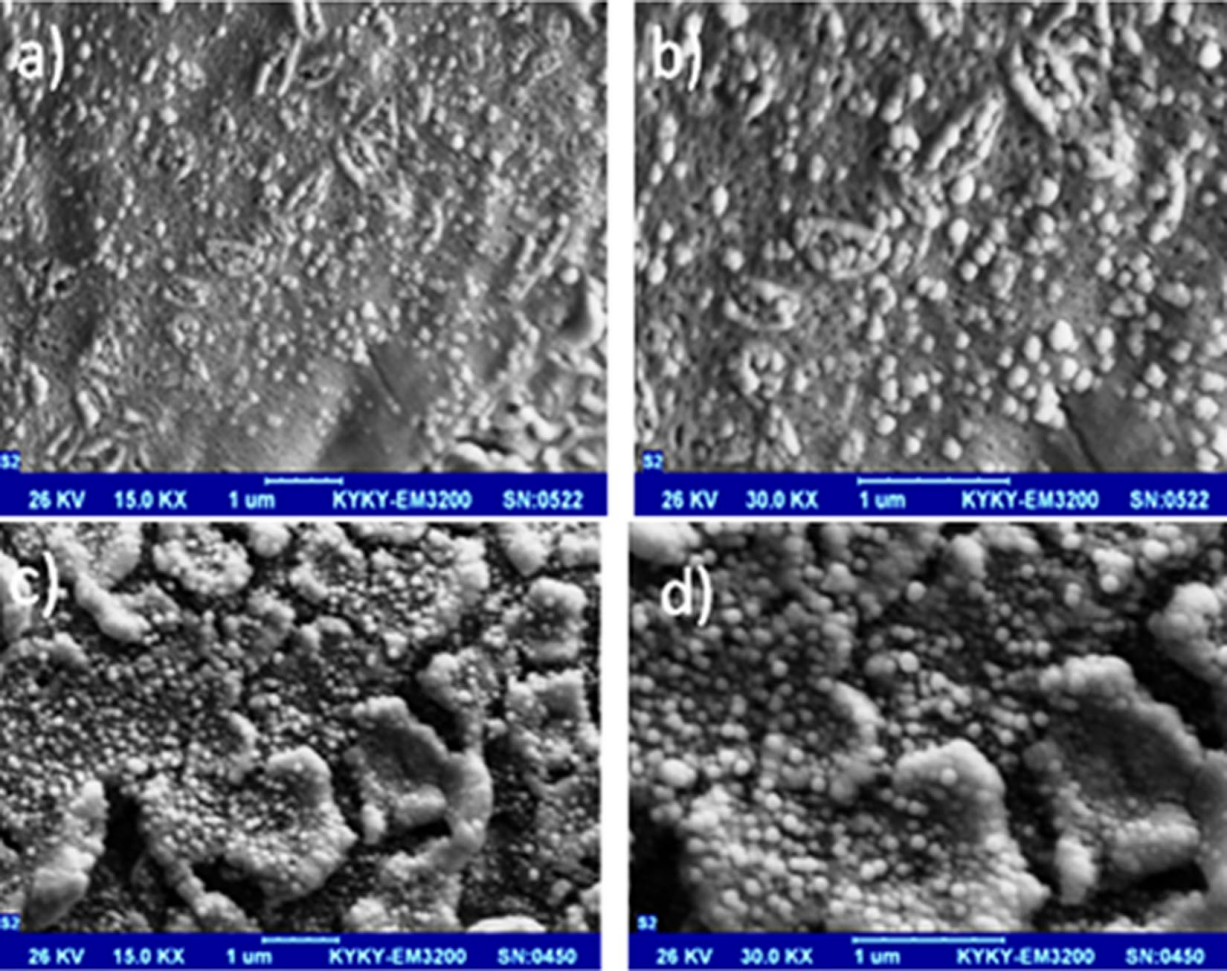
SEM micrograph of (**a**,**b**) C/S/N-doped Titania and (**c**,**d**) pure Titania with 15000 and 30000 magnification, respectively.

SEM micrographs of pure TiO_2_ and C/S/N-doped TiO_2_ photocatalysts are shown in Fig. 2. According to the micrographs, both samples consist of almost identical spherical particles. It can be concluded that dopants addition to TiO_2_ hindered the growth of TiO_2_ nanoparticles. Also, it is revealed that doping C, S, and N do not change the spherical shape of Titania samples and the porous surface.

**Figure 3:**
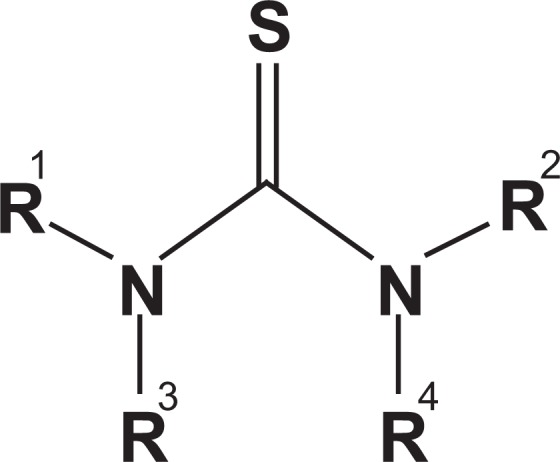
Thiourea as a flat three-ligand molecule that can interact with Titania unsymmetrical molecule using all three ligands especially through sulfur ligand^23^.

It seems adding C/S/N dopants postpone the nanoparticles aggregation. Thiourea is a three-ligand flat molecule which can interact with Titania unsymmetrical molecule using all the three ligands especially through sulfur ligand because of its stronger nucleophilic nature (Fig. 3)^23^. It may assumed that Thiourea is a corrosion inhibitor for Al mesh. Some research showed the inhibitor effect of Thiourea on Al through forming a protective film on the Al surface via adsorption^24^. Also doping of C, S, and N atoms could suppress the crystal growth of nanoparticles^25^.

### XRD results

**Figure 4:**
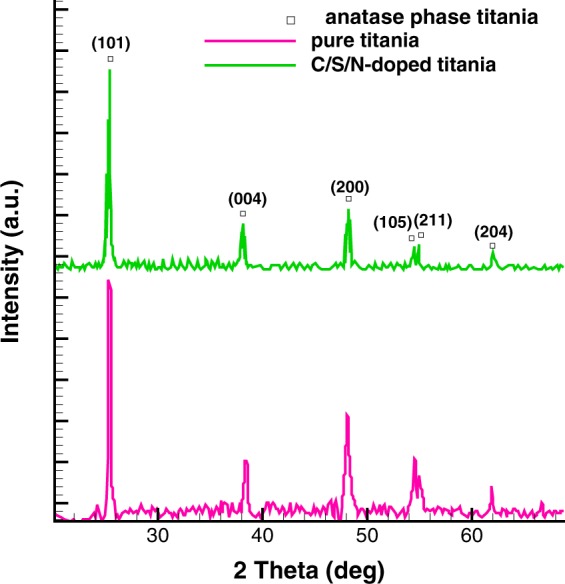
XRD pattern showing anatase phase of pure and C/S/N-doped TiO_2_.

The deposited layer scraped off from aluminum mesh to collect the powder for XRD characterization on zero background silicon. The background scatter from the substrate is close to zero in this method. The XRD patterns of pure and C/S/N doped TiO_2_ are shown in Fig. 4. It is observed that both samples exhibit well crystallized phase of anatase with the characteristic (101) plane^26^ with a small shift (about 0.029°) after doping which means that the crystal lattice are distorted by dopants. The average crystallite size of spherical particles was calculated through the width of diffraction peak (101) using Scherer’s equation:

1 $${\rm{D}}={\rm{k}}\,\lambda /\beta \,\cos \,{\rm{\theta }}$$

where D is the mean crystallite size (nm), λ is the wavelength of the Cu K_α_ X-ray radiation (λ = 0.15406 nm), k is a coefficient usually taken as 0.94, and β is the full width at half-maximum intensity of the diffraction peak (101) observed at 2θ. Also, the matrix distortion of pure and co-doped Titania lattices was estimated by XRD patterns using the following equation^27^:

2 $${\rm{\varepsilon }}={\rm{\beta }}/4\mathrm{tg}{\rm{\theta }}$$

The results of XRD analysis are summarized in Table 1. According to the results, nonmetal dopants resist against the aggregation of smaller crystallites, forming larger pores and surface areas.

**Table 1:** The XRD results for pure and codoped TiO_2_.

Sample	Mean crystallite size (nm)	Matrix distortion (%)
Pure Titania	12.9	1.27
C/S/N-doped Titania	10.4	1.58

Since the Al mesh has small cross section The XRD pattern was obtained using a powder XRD on zero background silicon. The background scatter from the substrate is close to zero in this method.

### EDX results

**Figure 5:**
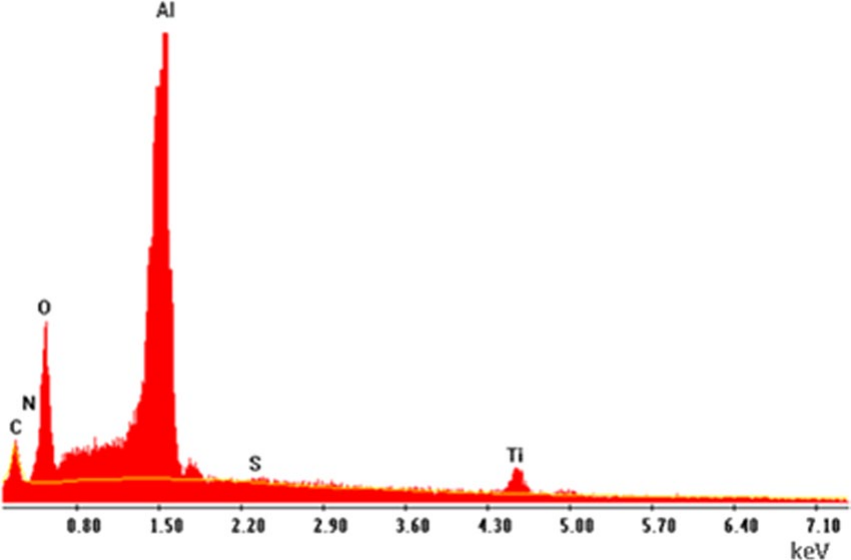
EDX pattern for C/S/N-doped TiO_2_.

EDX result of the coated aluminum mesh is given in Fig. 5 and is summarized in Table 2. Apparently, carbon atoms are well-substituted, while sulfur has the lowest ratio of both atomic and weight percent. As a result, the atomic ratio of Ti:O is about 3:25. The strong peak of the aluminum substrate is due to the low thickness of thin films.

**Table 2:** The EDX result for characterization of dopants weight and atomic ratio in co-doped sample.

Element	Weight percent (Wt%)	Atomic percent (At%)
Titanium	33.21	14.14
Oxygen	58.65	74.77
Carbon	3.00	5.09
Sulfur	1.81	1.15
Nitrogen	3.33	4.83

### UV-Vis results

**Figure 6:**
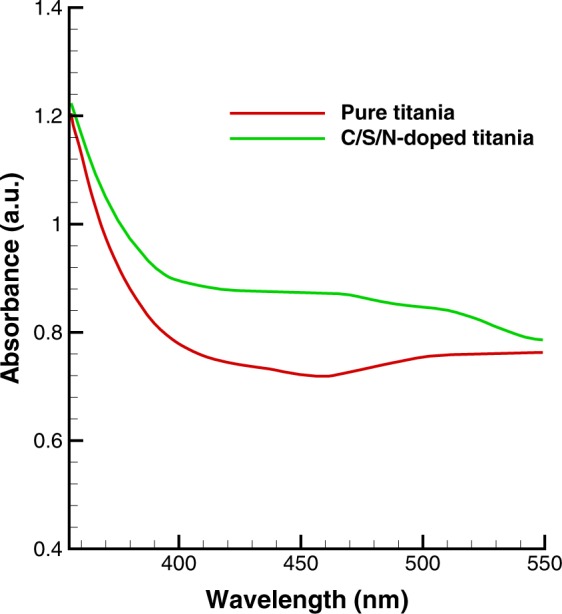
The absorption spectra of pure and C/S/N-doped TiO_2_.

**Figure 7:**
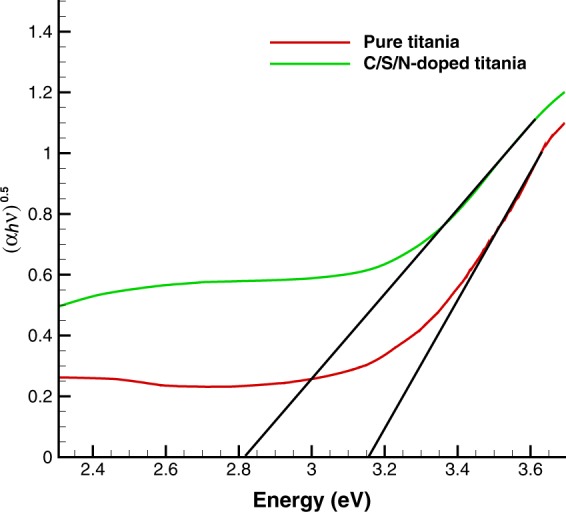
The plots of (*αhv*)^0.5^ versus energy (*hv*) for pure and C/S/N-doped Titania.

UV-Vis spectrophotometric analysis of C/S/N-doped Titania thin film and pure Titania are shown in Figs 6 and 7. The spectra of C/S/N-doped TiO_2_ sample exhibits considerable visible light region absorption. The band gap energies of pure and co-doped TiO_2_ are 3.18 and 2.80 eV, respectively. New energy levels of dopant species in the band gap of Titania increase the visible light absorption of the co-doped Titania. Actually, the p states of the nonmetal dopants (C, S, and N) form additional energy levels above the valence band or hybridize with 2p orbitals of O and lead to a decrease in the band gap of Titania and a strong redshift to the visible light region^28,29^.

### Photocatalytic activity measurements

Most of the VOCs like benzene degrade to water and carbon dioxide in presence of Titania and UV irradiation.

Benzene was selected for photocatalytic activity measurement. Six carbon atoms in Benzene compact very tightly. These atoms have six 2p hybrid orbital symmetry axis vertical to the plane of the ring, and overlap each other, forming a tight п bond. Electron cloud is shared equally in this п bond so it is hard to be attacked by free radicals and oxidants^30^.

**Figure 8:**
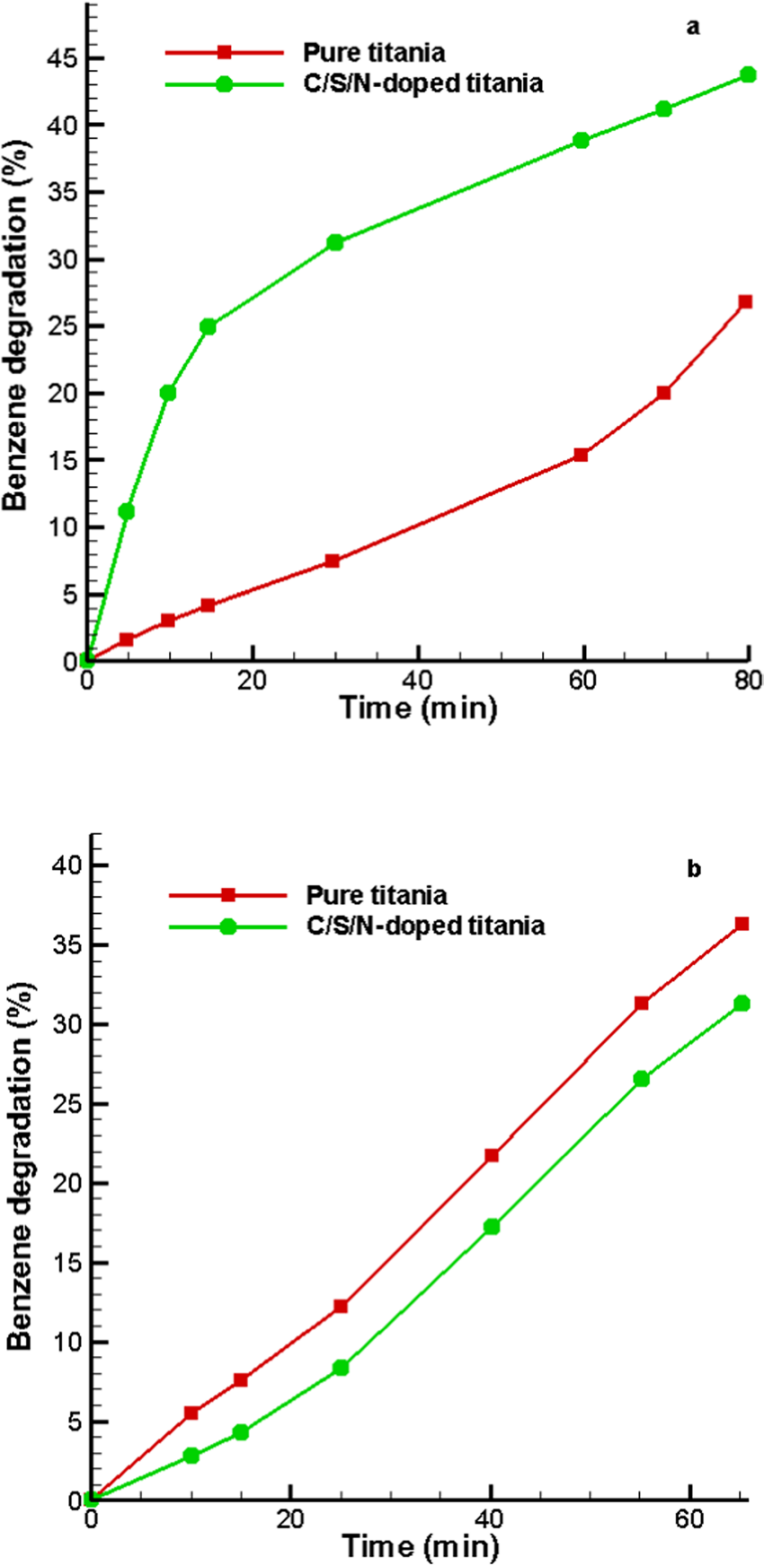
Photodegradation of benzene in presence of pure and C/S/N-doped Titania under (**a**) visible light and (**b**) UV light illumination.

The adsorption-desorption experiments were initially performed in darkness for 45 minutes, then the photocatalytic activity of pure and codoped-TiO_2_ were examined under UV and visible light sources, separately. As expected, the C/S/N co-doped Titania showed higher photocatalytic activity compare to pure Titania for degradation of benzene (Benzene concentration: 50 μg/lit) under visible light illumination. Photocatalytic activity of C/S/N co-doped samples and pure TiO_2_ under visible light source irradiation is shown in Fig. 8(a). It is seen that the pure TiO_2_ photocatalytic activity is considerably decreased under visible light illumination due to the lack of photogenerated charge carriers. Photocatalytic activity of both samples under UV source illumination is shown in Fig. 8(b). According to the results, the pure Titania showed higher activity than the co-doped one.

The improvement of photocatalytic activity of C/S/N co-doped TiO_2_ compared to pure TiO_2_ is regarded to its small crystalline size, intense light absorption in the visible region, large number of surface hydroxyl groups and low recombination rate of photogenerated charge carriers. Actually, C, N, and S dopants narrow the band gap of Titania and improve the visible light absorption.

In the case, the visible light photodegradation of benzene occurred as below: the co-doped catalyst was excited by visible light illumination and charge carriers were generated which could create oxidation-reduction reactions. The conduction band of electrons or trapped electrons reduced O_2_ molecules near the photocatalyst surface and produced superoxide radicals and the photo-induced holes created hydroxyl radicals from H_2_O molecules then the radical oxidizing species interacted with the benzene molecules and caused their degradation^31^.

### The reactor design

**Figure 9:**
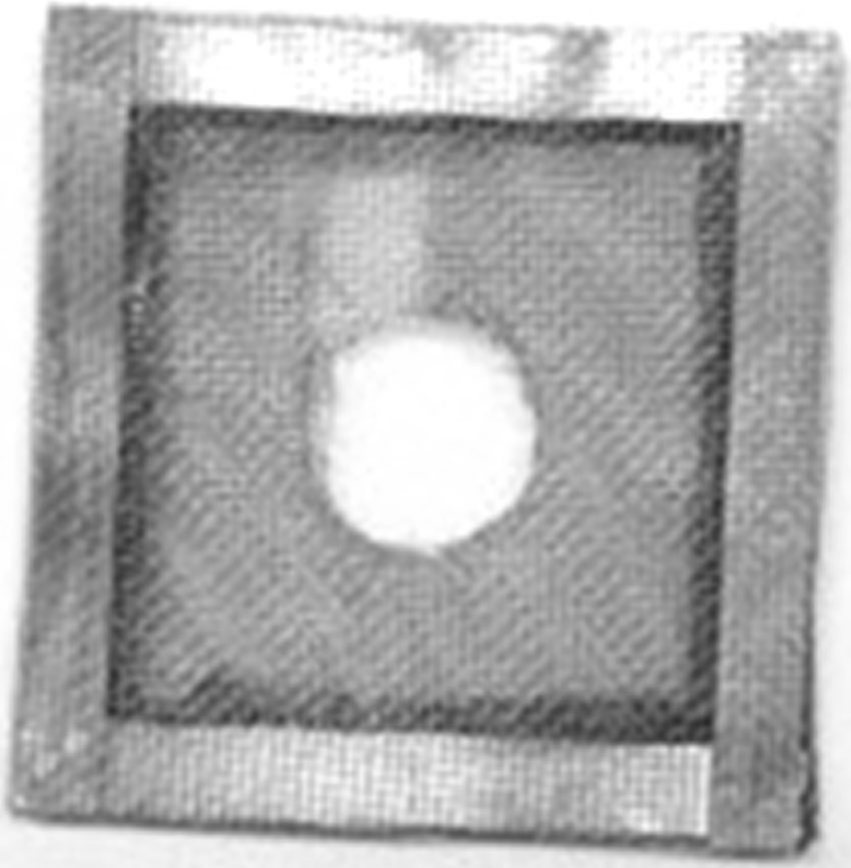
A sample of aluminum TiO_2_ coated mesh with aluminum frame and hole for UV lamp installation.

The novelty of this study is the reactors specific design. We employed Al mesh as thin film substrate for several reasons, first the large surface area of the mesh causes to increase specific surface area of the photocatalysts, it is noteworthy that, forming of shadows will stay in minimum level and the thin films will receive the highest irradiation of light source. Also, the low cost of Al meshes is one of the considerable advantages than the other kind of substrates (Fig. 9).

### The immobilization firmness

**Figure 10:**
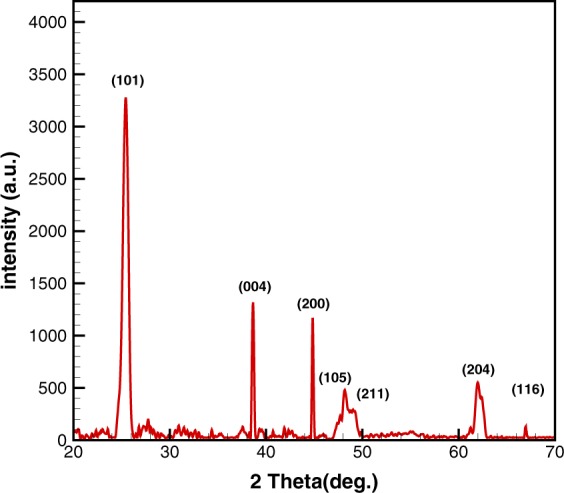
SEM image of C/S/N co-doped Titania after fifty times of using.

**Figure 11:**
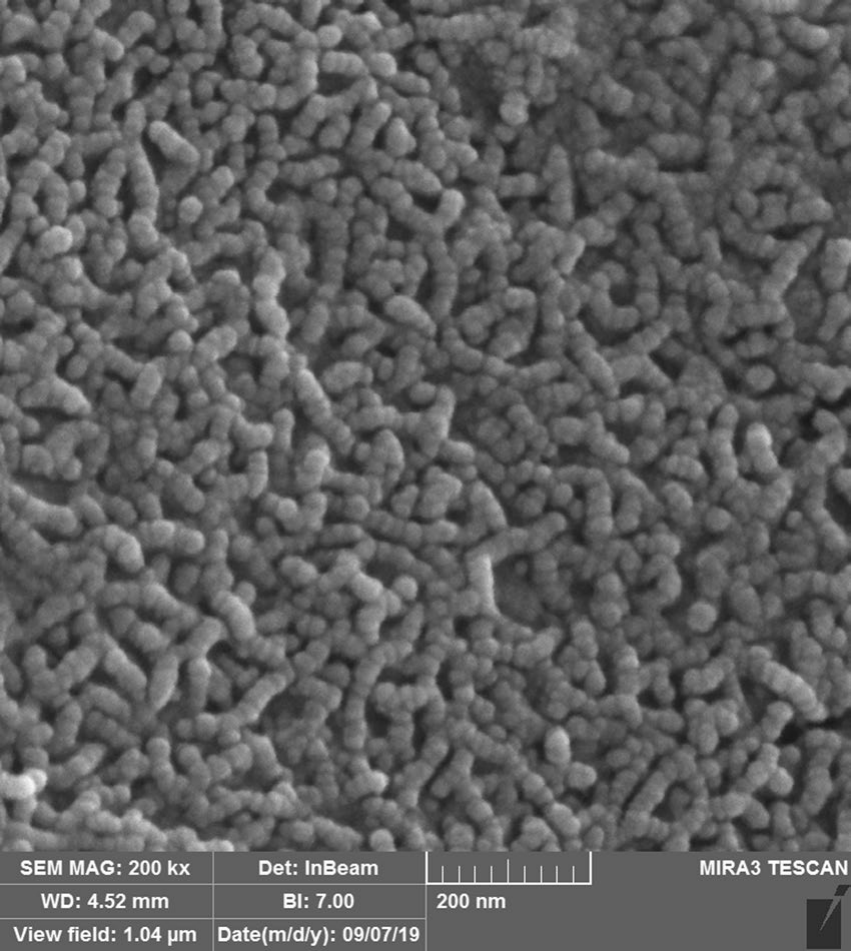
XRD pattern of C/S/N co-doped Titania after fifty times of operation.

The immobilization firmness of C/S/N co-doped Titania on Al mesh was evaluated through XRD and FESEM characterization. According to the FESEM image and XRD pattern, TiO_2_ nanoparticles didn’t eliminate or be washed out by air flows after running the photocatalyst system for more than fifty hours. The FESEM (TESCAN, MIRA3) and XRD (Rigaku, Ultima IV) have applied for these characterization (Figs 10 and 11). As the XRD pattern shows, the XRD peaks are as same as the former peaks.

## Conclusion

Pure and C/S/N doped TiO_2_ catalysts were prepared via a sol–gel method. XRD patterns showed the formation of anatase phase in all cases with a decrease in crystallite size of samples via co-doped modification. According to XRD data, both pure and co-doped Titania had a distortion in their crystal lattices. Moreover, UV-Vis spectra exhibited a redshift in the absorption edge of the co-doped photocatalyst toward the visible range. Photocatalytic measurements showed higher photocatalytic activity of co-doped TiO_2_ compared to pure TiO_2_ in benzene photo-degradation under the visible light illumination due to the synergistic effect of dopants incorporating to the Titania lattice. It can be assumed that the obtained photocatalysts had a better efficiency for removing many other VOCs which might show higher photocatalytic activity through further modification. Also, the appropriate design of the reactors in this work can assume as a promising method for future commercial efforts.

It is noticeable that just a few of too many indoor air treatment projects could pass the manufacturing issues and become commercialized, so the applied materials have to be available and also affordable. In this research we put our attempt into finding a new formable substrate with high surface area.

As a future prospective, this substrate can be engineered geometrically to reach to a minimum pressure drop of air flow while it is installed in air conditioners or HVAC systems.
